# Transportation Stress Increases Fos Immunoreactivity in the Paraventricular Nucleus, but Not in the Nucleus of the Hippocampal Commissure in the Pekin Duck, *Anas platyrhynchos domesticus*

**DOI:** 10.3390/ani12223213

**Published:** 2022-11-19

**Authors:** Sara Tonissen, Victoria Tetel, Gregory S. Fraley

**Affiliations:** Department of Animal Sciences, Purdue University, West Lafayette, IN 47907, USA

**Keywords:** sex difference, cortisol, corticosterone, nucleus of hippocampal commissure, paraventricular nucleus

## Abstract

**Simple Summary:**

Transportation is generally believed to be a stressful event in the lives of poultry; however, studies have shown conflicting effects of transportation on stress hormone release. The hormonal stress response in animals is the organism’s attempt to maintain physiological homeostasis for survival. The systems involved in maintaining physiological homeostasis for survival include the brain, the pituitary and adrenal glands, and is referred to as the HPA axis. In birds, the HPA axis is different than it is in mammals, and may involve two adrenal hormones, corticosterone and cortisol. The purpose of our study was to investigate how transportation affected the release of both of these hormones, as well as transportation effects on brain areas that initiate the HPA axis. Our results suggest that simply measuring the adrenal hormones may be insufficient to determine a bird’s welfare status, and that stressors may have longer-term effects on the brain than previously believed. Thus, care must be taken on farms to prevent other stressors from occurring after transportation so as not to adversely affect the birds’ health, welfare, and production.

**Abstract:**

Commercial poultry undergo transportation during their life, and the effects of transportation can negatively impact poultry production and welfare. In order to maintain physiological homeostasis, the hypothalamic–pituitary–adrenal axis (HPA) works to respond to stressors. Previous studies by others have shown contradictory effects of transportation on corticosterone release. However, recent studies from our lab and by others have shown that cortisol may also be an important hormone in the avian HPA. The purpose of our current study was to determine the effects of transportation stress on the stimulation of brain nuclei that regulate the HPA in birds, and on glucocorticoid (GC) secretion. To test this hypothesis, we collected blood and brain samples from developer drakes and hens (N = 10 per sex/time point): 24 h prior to transportation, immediately after transportation, 24 h after transportation, and 1 week after transportation. Serum GC levels and fos immunocytochemistry (ICC) within the nucleus of the hippocampal commissure (NHpC) and paraventricular nucleus (PVN) were measured. Data were analyzed using a two-way ANOVA. Post hoc analysis was completed using a Fisher’s PLSD with a *p* < 0.05 considered significant. We observed a sex difference (*p* < 0.05) in both corticosterone and cortisol secretion in Pekin ducks, although neither GC showed a significant increase in secretion associated with transportation. However, we did observe a significant (*p* < 0.05) increase in fos-like immunoreactivity for 24 h in the PVN, but not in the NHpC. Further studies are required to determine the specific role that GCs play in the avian stress response and the short-term stressors that could have long-term physiological effects on birds.

## 1. Introduction

The hypothalamic–pituitary–adrenal axis (HPA) works as a central response to stressors in order to maintain physiological homeostasis. It has been determined that the HPA works very differently in avians compared to mammals. The central component of the HPA is comprised of two neuropeptides released from the median eminence: corticotrophin-releasing hormone (CRH), primarily found in the nucleus of the hippocampal commissure (NHpC) in the thalamus, and arginine vasotocin (AVT), primarily found in the paraventricular nucleus (PVN) within the hypothalamus [1,2]. However, both neuropeptides can be found in both nuclei. It is generally believed that the NHpC in chickens initiates the stress response, while the PVN is responsible for maintaining the stress response [3,4]. The relative activation of these two brain nuclei in the duck following stress is currently unknown, but is the primary focus of this report. Adrenocorticotropic hormone (ACTH), which is secreted by the anterior pituitary gland, and the glucocorticoid (GC) hormone corticosterone, which is synthesized by the adrenocortical cells, also play roles within the HPA [1]. However, original studies suggested that birds also secrete the glucocorticoid cortisol along with corticosterone. Kalliecharan (1981) demonstrated that ACTH did not selectively act on either the corticosterone or cortisol synthetic pathways in immature chicks, but rather increased both glucocorticoids [5]. Zenoble et al. showed that exogenous ACTH stimulated cortisol in parrots; however, this observation has not been replicated in other psittacines [6,7]. Little is known about the effects of cortisol in birds, but there is current evidence that it may play an important physiological role within avian species [8,9,10,11], including the duck [12]. It is important to better understand the full stress response associated with the HPA to be able to assess the welfare of any avian species.

Commercial meat duck welfare and overall production can be negatively impacted by a diverse array of environmental factors, including transportation stress. Effects of transportation should be taken into consideration within the poultry industry because commercial poultry are submitted to multiple instances of transportation throughout their lives. In the duck industry, commercial meat ducks may be subjected to transportation throughout three stages of their life: from hatchery to brooder houses, from developer to breeder houses, and from breeder houses to processing [13,14]. Factors such as stocking density, transportation duration, truck microclimate, overall animal health, and the quality of management practices can impact welfare and meat quality in livestock as well as commercial poultry [15]. Duration of transport and the microclimate within the trailer are two factors that are important to consider while transporting poultry. These factors have been shown to negatively impact core body temperature, physiology, and behavior in layer pullets and end-of-cycle hens [16,17]. Numerous past studies that measured corticosterone responses to transportation have resulted in conflicting reports, with some showing increased corticosterone, others decreased, and still others no effects at all (for review, see [1]). No studies have assessed cortisol secretion associated with transportation. When studying the factors that impact Pekin duck welfare, such as transportation, it is important to look at how the brain contributes to the avian stress response in relation to both corticosterone and cortisol release.

The purpose of our study was to determine the effects of transportation on stimulating brain nuclei that regulate the HPA in birds, and on GC release. To accomplish this goal, we measured serum corticosterone and cortisol levels as well as fos immunocytochemistry (ICC) in brain areas known to be involved in the avian HPA. We determined that there is a sex difference in GC secretion in Pekin ducks and that transportation may have central effects that last for 24 h after transportation. We also saw an increase in fos-like immunoreactivity for 24 h in the PVN, but not in the NHpC, in a sex-dependent manner. Our data suggest that certain stressors, such as transportation, could have physiological effects that last for 24 h or more in birds.

## 2. Materials and Methods

### 2.1. Experimental Design

The transportation segment of the experiment was performed onsite at Maple Leaf Farms. We followed an identical protocol as previously reported in our lab [12]; however, this study was completed at a different time on different ducks from different barns than in that study. In brief, the ducks were transported from the developer barn to the breeder barn with an approximated travel time of 1 h. Developer (14 weeks of age) drakes and hens were assessed 24 h before transportation (Pre-transport), as they walked off the truck (Transport), 24 h after transportation (Post-transport + 24), and 1 week after transportation (Post-transport + 1 week; N = 10 per sex/time point). During this process, ducks are slowly and gently herded down the length of the developer barn to walk up a ramp in order to load onto the semi-trailer poultry transport. On the trailer, ducks are placed at a density of about 0.05 m^2^/duck. After an approximately 1 h drive, the trailer barriers are opened and the ducks are allowed to walk down the ramp to enter the new breeder barn. For the Transport group, blood and brains were collected from the first ducks off the truck at the breeder barn. Ducks were picked up, and blood was collected from the tibial vein into serum separator tubes. The tubes were then centrifuged, and the serum was stored at −20 °C until used in the ELISAs. Ducks were immediately euthanized using intravenous FatalPlus (pentobarbital, 396 mg/mL/kg). The time from blood collection to injection of FatalPlus for each bird took less than 2 min. Brains were collected from euthanized ducks at each time point and placed into 4% paraformaldehyde (pH = 7.4) for 72 h, then into cryopreservative (25% sucrose in 0.1 M phosphate buffer, pH = 7.4; PB) until the brains sank. Coronal sections (40 μm) were collected into 4 equal sets from the septomesencephalic tract to the third cranial nerve, thus spanning the diencephalon and the NHpC. Sections were stored in antifreeze (cryoslime; 40% ethylene glycol, 30% sucrose in 0.1M phosphate buffer, pH = 7.4) at −20 °C until they were processed for immunocytochemistry (ICC). All experiments were approved by the Purdue University Animal Care and Use Committee (PACUC #2008002065).

### 2.2. ELISA Assay for Glucocorticoids

The kits utilized for this project were from Cayman Chemicals and the assays were run according to the manufacturer’s recommendations. Details of the kit verification by our lab have been reported previously [12]. Samples were run in undiluted duplicates. Plates were incubated with samples overnight at 4 °C and, after development, were read at 405 nm (SynergyLx, Biotek).

### 2.3. Fos Immunocytochemistry (ICC)

The ICC protocol was followed as previously published by our lab using the free-floating section method [18,19,20,21,22]. Brain sections were collected from 10 drakes and 10 hens during each sampling time: Pre-transport 24 h, Transport, Post-transport + 24 h, and Post-transport + 1 week. One set of free-floating sections was allowed to come to room temperature, then washed in 100 mL of 1× PB for three rounds at 10 min each using a platform shaker. The sections were then transferred to a 1:10 dilution of hydrogen peroxide in double-distilled water and incubated for 20 min in order to remove endogenous peroxidase activity, followed by 4 rounds of washes in 1× PB for 10 min each. To improve antigen retrieval, sections were incubated in 100 mL of sodium citrate (10 mM) that was heated to 90 °C and allowed to cool to room temperature (~30 min) during the incubation with the tissues. The sections were then washed in 1× PB for four rounds at 10 min each. Finally, the sections were incubated with an anti-fos rabbit polyclonal antibody (Abcam 1:2000 dilution) in 1× PB with 0.03% Triton-X 100. The sections were incubated on a shaking table for 1 h at room temperature, then at 4 °C for 48 h.

After 48 h, the sections were washed in 1× PB for four rounds at 15 min each. The sections were then incubated for 1 h at room temperature in 1× PB and 0.04% Triton-X 100 with 1:500 biotinylated-horse-anti-rabbit IgG antibody (Vectqastain, Vector Labs, Inc., Newark, CA, USA) on a shaking table. The sections were again washed in 1× PB for 3 rounds at 15 min each. Sections were then incubated in an avidin–biotin complex (ABC, Vectastain, Inc.) per the manufacturer’s recommendation for 30 min at room temperature. After washing, the chromogen reaction was conducted again per the manufacturer’s recommendation using DAB in the presence of NiCl_2_ and hydrogen peroxide. The sections were then washed in 1× PB for 4 rounds at 2, 5, 10, and 15 min, dehydrated in serial ethanol, defatted in Histoclear, and coverslipped using DPX mounting media. Elimination of either the primary or secondary antibody prevented all staining.

All slides were coded then randomized for analyses. The person responsible for fos-like immunoreactivity (-ir) counts was unaware of the treatment groups. The microscope slides were viewed under a brightfield microscope at 200× and the fos-ir nuclei were counted in the paraventricular nucleus (PVN) and the nucleus of the hippocampal commissure (NHpC). All dark blue-black nuclei were counted as positive, and fos-ir were counted bilaterally on all sections that contained one or both nuclei. Fos-ir counts were analyzed as total numbers per neuronal nucleus.

### 2.4. Statistical Analyses

The duck was considered the statistical unit throughout this experiment. Due to the use of separate ELISA kits, cortisol and corticosterone were not analyzed against each other. Statistical analysis for both hormone and fos analyses was completed using a 2-way ANOVA (sex × time). Post hoc analysis was completed using a Fisher’s PLSD with a *p* < 0.05 considered significant.

## 3. Results

### 3.1. Effects of Transportation Stress on Serum Glucocorticoids

**Figure 1:**
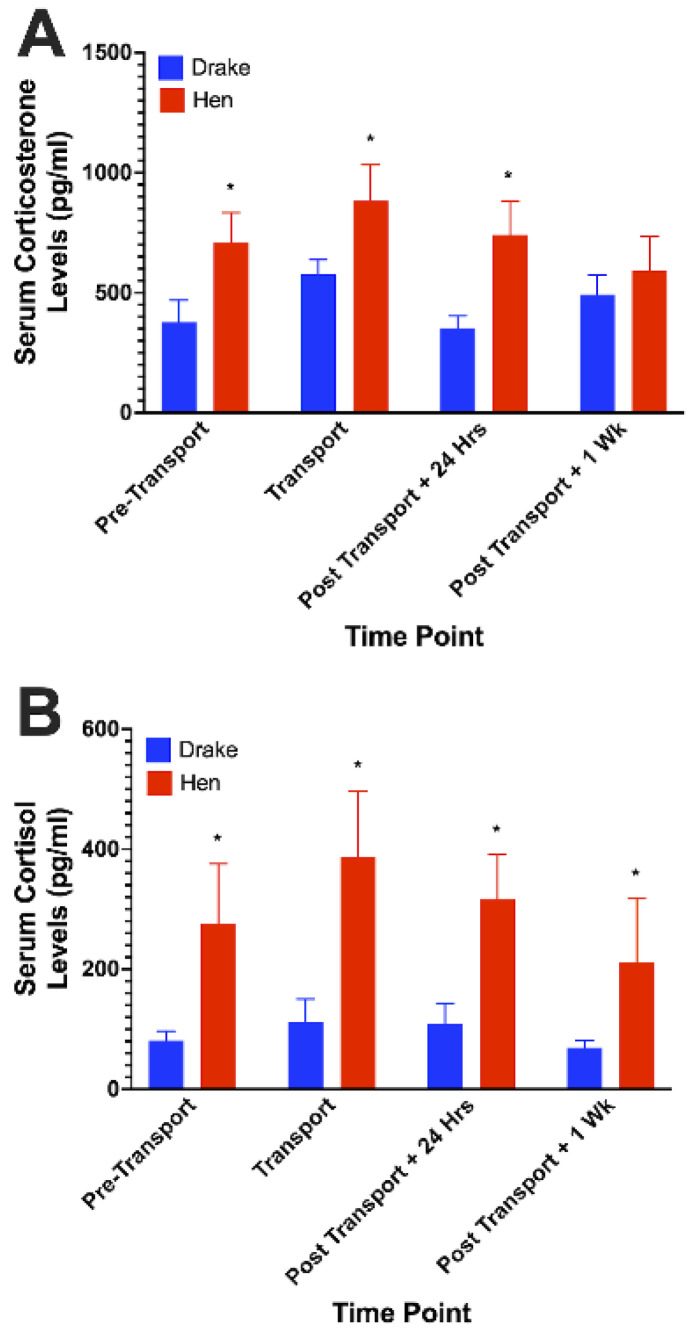
Serum glucocorticoids in ducks following transportation stress. Sex differences were observed in that hens showed significantly greater levels of both circulating corticosterone (**A**) and cortisol (**B**) compared to drakes. Transportation elicited a non-significant increase in both glucocorticoids. The lack of significant increase may likely be due to the timing of blood sampling relative to the perceived onset of stress. * = *p* < 0.05.

For all time points except 1 week Post-transport, hens showed significantly (F_1,72_ = 12.87; *p* < 0.01) higher levels of corticosterone in their serum compared to drakes. At all timepoints, hens showed significantly (F_1,72_ = 16.06; *p* < 0.05) higher levels of cortisol than drakes. Both glucocorticoids showed a slight, but non-significant increase in the serum of hens and drakes in association with transportation. Results are illustrated in Figure 1.

### 3.2. Fos Immunocytochemistry (ICC)

**Figure 2:**
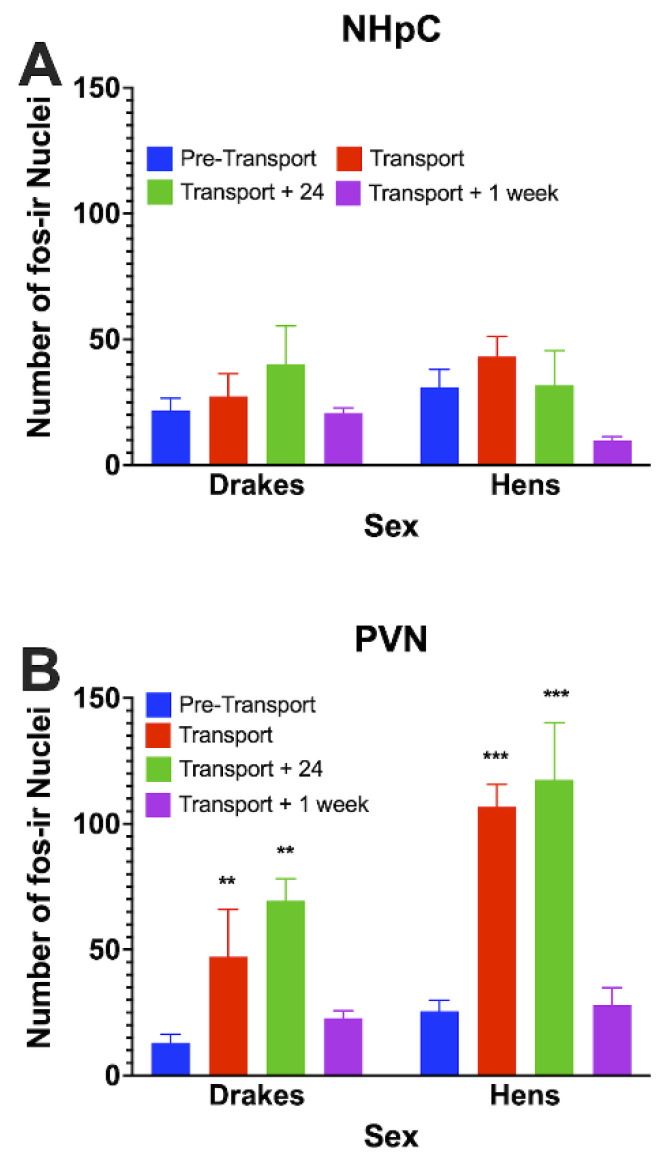
Fos-ir in the NHpC and PVN associated with transportation. A significant increase in the number of fos-ir was observed at Transportation in the PVN (**B**) but not in the NHpC (**A**) that lasted through the Transportation + 24 h sample time. Further, a significant sex difference was observed in that hens showed a greater number of fos-ir in the PVN compared to drakes at Transportation and Transportation + 24 h. Asterisks indicate differences from controls of the same sex where, ** = *p* < 0.01, *** = *p* < 0.001. Differences between sexes at same time points, *p* = 0.0036.

**Figure 3:**
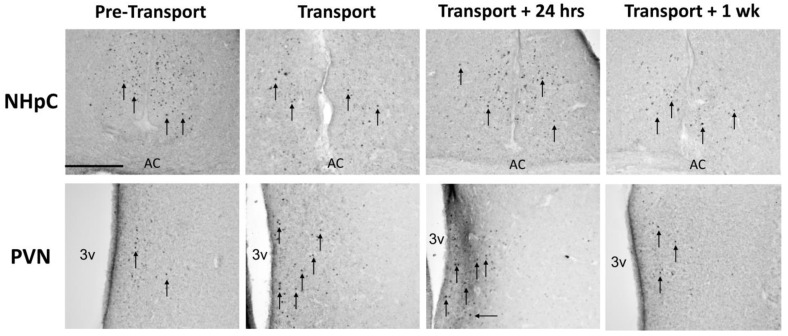
Representative photomicrographs of fos-ir. Top row: no differences in fos-ir were observed in the NHpC at any time point assessed. Bottom row: the number of fos-ir nuclei in the PVN increased with Transportation and continued to show increased numbers for 24 h before returning to Pre-transport levels. Arrows = fos-ir nuclei. AC = anterior commissure. 3v = third ventricle. Bar = 100 μm. Arrows point to representative fos-ir.

We observed that transportation affected one of the brain areas that regulate the HPA in ducks. The number of fos nuclei present in the PVN, but not in the NHpC, significantly (F_2,14_ = 18.72; *p* < 0.01) increased between the Pre-transport and Transport time periods. The significant increase in fos-ir in the PVN persisted 24 h after transportation, then returned to control levels within 1 week following transportation. At the Transportation and Post-Transport + 24 h time points, hens showed a significantly (F_1,8_ = 16.6; *p* = 0.0036) greater number of fos-ir in the PVN than drakes. Results are illustrated in Figure 2. Representative photomicrographs are presented in Figure 3.

## 4. Discussion

The purpose of this study was to determine whether transportation stimulates brain nuclei known to initiate the hypothalamic–pituitary–adrenal axis (HPA) in birds. To accomplish this goal, we collected brains from adult ducks prior to, during, and at two time points following transportation. Brains were processed for immunocytochemistry for fos-immunoreactivity (-ir). Blood samples were also collected for glucocorticoid (GC) analyses at the same time points. Analyses showed that around the time of transport we observed a trend toward increasing levels of corticosterone and cortisol in the serum of both hens and drakes. However, neither hormone showed a significant increase over time, similar to our previous study [12]. The lack of significant increase in glucocorticoids is likely due to variability in the timing of the blood collection relative to when the individual duck perceived the onset of the stressor [12,23]. The effects of transportation on GC levels in ducks has been described in a previous report by our lab [12]. Surprisingly, we did observe significantly increased levels of both glucocorticoids in hens compared to drakes at nearly every time point. Thus, sex differences in the HPA response of ducks have become a consistent finding in our lab, including in the current report [12,23]. Most importantly, we observed a significant increase in fos-ir in the paraventricular nucleus (PVN), but not in the nucleus of the hippocampal commissure (NHpC), that lasted for 24 h following transportation. We are not able to determine whether this prolonged fos activation is due solely to the transportation, or due to combined effects of transportation with the new barn environment, or due to potential social stressors associated with both states. However, a sex difference was also observed in that hens showed a greater increase in fos-ir than drakes as a result of the transportation. Our results suggest that short-term stressors may have longer-lasting consequences on the brain, resulting in other potential physiological ramifications that need to be considered when addressing the stress or welfare states of birds.

In order to study the long-term neurological effects of stressors, it is important to understand the roles of the NHpC and the PVN in the HPA and the stress response in birds. In the mammalian HPA, corticotropin-releasing hormone (CRH) and vasopressin (VP) are two important neuropeptides that contribute to the stress response, although VP does so only under periods of extreme dehydration [24]. However, in birds, arginine vasotocin (AVT), a substance that has a single amino acid substitution compared to arginine vasopressin found in mammals, is synthesized and used in the HPA [3]. Kuenzel et al. (2020) suggested that CRH caused a stronger release of adrenocorticotropic hormone (ACTH), but AVT triggered a more effective release of ACTH. A study by Kadhim et al. (2019) found that feed deprivation rapidly increased CRH levels within the NHpC before returning to baseline levels. Concurrently, levels of CRH in the PVN increased at a constant rate throughout a 4 h time period following food deprivation. In that same Kadhim et al. (2019) study, they also found that corticosterone levels increased significantly during the second hour of the experiment and persisted throughout, suggesting that the NHpC initiated the release while the PVN sustained the stress response [4]. Another study supports these data through the findings that CRH genes were rapidly activated in the NHpC compared to the PVN after a 4 h feed deprivation [25]. The differences in the roles between the NHpC and the PVN help to elucidate how stressors can cause long-term neurological effects. Our fos-ir data further support the idea that the PVN may have a longer-term effect following a stressor compared to the NHpC, and that the NHpC may initiate the stress response, but the PVN maintains this response, as suggested by others [3]. It would have been valuable in our current study to have colocalized fos-ir with either AVT or CRH; however, those primary antibodies do not work in ducks, at least not without the use of cholchicine, which is well known for altering fos-ir, thus confounding interpretation. Regardless, data from our current study and past studies have shown that taking the bird’s sex into consideration is critical when evaluating HPA function [12,23].

Along with differences in NHpC and PVN activation, sex differences affect the overall function of the HPA in avian species. Madison et al. (2018) studied social stress between separated pairs of mated zebra finches to measure circulating corticosterone concentrations along with gene expression levels of mineralocorticoid receptors (MR) and glucocorticoid receptors (GR) within the hippocampus. Their study observed that females showed an upregulation of MR mRNA within the hippocampus while males showed a downregulation of MR and GR mRNA [26]. Jimeno et al. (2017) manipulated brood size (small and large) and foraging conditions (easy or difficult) and measured baseline and stress-induced corticosterone levels in zebra finches. Their study found that male baseline corticosterone levels were not affected by the different treatments. However, Jimeno et al. (2017) observed that female baseline corticosterone levels were higher after being reared in large broods and subjected to difficult foraging conditions as adults [27]. It is interesting that others have shown that domesticated chickens have a reduced HPA response compared to their wild red junglefowl counterparts [28]. Further, domestication may have altered gene expression following stressors such as feed restriction, including CRH and AVT, and fos expression in the pituitary gland [28]. Chickens have been selectively bred for decades to withstand stressors, such as those in conventional cages, while domestic ducks have not had similar selective pressures placed on them [13]. Currently, the distribution of CRH and AVT in the duck is unknown, so a comparison of the fos-ir distribution in the current study cannot be made. Further, future studies should continue to determine the extent of the role that sex differences play in the HPA of birds, and how these differ between domestic and wild counterparts.

Schmidt et al. (2008) performed a study on developing zebra finches and found that, while corticosterone is the predominant glucocorticoid within plasma, cortisol was more predominant in immune tissues. Moreover, this study found that corticosterone levels tended to increase with age while cortisol levels tended to decrease with age, suggesting that age is also an important factor related to stress [8]. Our lab has previously shown that both corticosterone and cortisol were found in duck serum in response to shipping stress [12], there is a sex difference that occurs in response to ACTH stimulation, and there are subsequent changes in heterophil to lymphocyte ratios (HLR) in the duck [23]. 

## 5. Conclusions

Ours is the first study to show a long-term increase in fos-ir following a relatively short-term stressor. Our study provides further evidence that the NHpC may be responsible for initiating the endocrine response to stress, while the PVN maintains that response. Further studies are needed to determine the specific role of cortisol in the avian stress response and the ramifications of the long-term activation of neurons within the PVN. 

## Data Availability

The data presented in this study are available on request from the corresponding author.
